# Phagocytosis and LPS alter the maturation state of β-amyloid precursor protein and induce different Aβ peptide release signatures in human mononuclear phagocytes

**DOI:** 10.1186/1742-2094-7-59

**Published:** 2010-10-07

**Authors:** Philipp Spitzer, Martin Herrmann, Hans-Wolfgang Klafki, Alexander Smirnov, Piotr Lewczuk, Johannes Kornhuber, Jens Wiltfang, Juan Manuel Maler

**Affiliations:** 1 Department of Psychiatry and Psychotherapy, University of Erlangen-Nuremberg, Schwabachanlage 6, D-91054 Erlangen, Germany; 2 Department of Medicine III, Institute for Clinical Immunology, University of Erlangen-Nuremberg, Glückstr. 4a, D-91054 Erlangen, Germany; 3 Department of Psychiatry and Psychotherapy, University of Essen, Virchowstraße 174, D-45147 Essen, Germany

## Abstract

**Background:**

The classic neuritic β-amyloid plaque of Alzheimer's disease (AD) is typically associated with activated microglia and neuroinflammation. Similarly, cerebrovascular β-amyloid (Aβ) deposits are surrounded by perivascular macrophages. Both observations indicate a contribution of the mononuclear phagocyte system to the development of β-amyloid.

**Methods:**

Human CD14-positive mononuclear phagocytes were isolated from EDTA-anticoagulated blood by magnetic activated cell sorting. After a cultivation period of 72 hours in serum-free medium we assessed the protein levels of amyloid precursor protein (APP) as well as the patterns and the amounts of released Aβ peptides by ELISA or one-dimensional and two-dimensional urea-based SDS-PAGE followed by western immunoblotting.

**Results:**

We observed strong and significant increases in Aβ peptide release upon phagocytosis of acetylated low density lipoprotein (acLDL) or polystyrene beads and also after activation of the CD14/TLR4 pathway by stimulation with LPS. The proportion of released N-terminally truncated Aβ variants was increased after stimulation with polystyrene beads and acLDL but not after stimulation with LPS. Furthermore, strong shifts in the proportions of single Aβ_1-40 _and Aβ_2-40 _variants were detected resulting in a stimulus-specific Aβ signature. The increased release of Aβ peptides was accompanied by elevated levels of full length APP in the cells. The maturation state of APP was correlated with the release of N-terminally truncated Aβ peptides.

**Conclusions:**

These findings indicate that mononuclear phagocytes potentially contribute to the various N-truncated Aβ variants found in AD β-amyloid plaques, especially under neuroinflammatory conditions.

## Background

The neuropathological changes typically found in Alzheimer's disease (AD) brains include the formation of neurofibrillary tangles, the deposition of multiple Aβ peptide variants into neuritic β-amyloid plaques and neuroinflammation. Neuritic plaques are complex lesions which vary in their morphology and Aβ peptide composition [1]. Classic neuritic plaques consist of a dense β-amyloid core which is surrounded by elevated numbers of activated microglial cells and paired helical filament-type dystrophic neurites [2]. Aβ peptides ending at valine-40 (Aβ40) are highly abundant in cored plaques while diffuse Aβ plaques consist mainly of longer peptides ending at alanine-42 (Aβ42) [3]. It is assumed that microglia is involved in the maturation of plaques, especially in the deposition of Aβ40 [4]. Similarly, cerebrovascular β-amyloid deposits that are also found in AD are surrounded by perivascular macrophages [5]. Both of these plaque-associated cell types belong to the mononuclear phagocyte system and are partly recruited from blood [6]. In addition to Aβ_1-40 _and Aβ_1-42 _several other Aβ species, especially N-truncated forms, were detected in neuritic and vascular β-amyloid plaques. N-truncated Aβ peptides are present in the earliest stages of AD pathology and their proportion in β-amyloid deposits increases along the course of the disease correlating with the Braak stage [7,8]. N-truncated Aβ peptide variants account for more than 60% of all Aβ peptides found in cored plaques at Braak stage VI [8,9]. However, neither their origin nor their role in amyloidogenesis is fully understood.

It is also not clear whether activated microglia contributes to the formation of neuritic plaques by defective phagocytosis of Aβ or by the *de novo *production and subsequent deposition of Aβ peptides [10]. *De novo *production and subsequent release of Aβ results from the proteolytic processing of amyloid precursor protein by β- and γ-secretases in distinct cellular compartments [11]. While APP695 is predominantly expressed by neurons, the longer KPI-containing isoforms APP751 and APP770 are more abundantly expressed by microglia and blood mononuclear cells [12-14]. APP undergoes N- and O-glycosylation during maturation in the endoplasmic reticulum and the Golgi network [15]. The glycosylation state was shown to influence the transport of APP to distinct cellular compartments and thereby its proteolytic processing [15-17].

Several surface receptors were reported to be involved in phagocytosis and the interaction of microglia/mononuclear phagocytes with fibrillar Aβ or β-amyloid plaques. Scavenger receptors are strongly expressed in association with senile plaques in AD [18]. Both class A (SR-A) and class B scavenger receptors (SR-B) are expressed on macrophages and macrophage-derived cells and are activated by acetylated low density lipoprotein (acLDL), oxidized LDL (oxLDL), advanced glycation endproducts (AGE) and by phagocytosis of polystyrene beads [19,20]. They are also the main receptors mediating the microglial activation by fibrillar Aβ and endocytosis of fibrillar Aβ by microglia [21,22]. Furthermore, the expression of the lipopolysaccharide (LPS) receptor (CD14) is increased on cortical and hippocampal microglia in AD and in primary murine microglial cells fibrillar Aβ_1-42 _is phagocytosed in a CD14-dependent manner [23].

We have previously investigated the Aβ peptides released by human mononuclear phagocyte cultures as a model for microglia. We observed that their activation by adherence to polystyrene surfaces induced an overall increase of Aβ peptide release and a relative increase in N-terminally truncated Aβ species [24]. The objective of the present study was to investigate the influence of plaque-associated inflammatory events such as phagocytosis and activation of the LPS receptor on the secretion of Aβ peptides by human mononuclear phagocytes.

## Methods

### Monocyte isolation and culture

EDTA-anticoagulated blood was obtained by venipuncture from young, non demented indi-viduals. Signed consent was obtained from all volunteers and the study procedures were approved by the ethics committee of the University of Erlangen-Nuremberg. Peripheral blood mononuclear cells (PBMC) were isolated by Ficoll centrifugation as detailed previously [24]. Afterwards, CD14 positive mononuclear phagocytes were selected by antibody mediated removal of non-monocytes by magnetic activated cell sorting (MACS) according to the manufacturers protocol (Monocyte Isolation Kit II, Miltenyi Biotech, Germany). The purity of the negatively selected monocyte fraction was ≥90%, while CD61 positive thrombocytes accounted for only ~3% of total cell counts as assessed by flow cytometry (data not shown). Cells were resuspended in serum-free (Aβ-free) AIM-V medium, and seeded at a density of 2 × 10^6^/ml in 24-well ultra low binding plates (Corning, The Netherlands). Mononuclear phagocytes were stimulated with LPS 10 ng/ml (from E. coli serotype O111:B4; Sigma-Aldrich, Germany), carboxylated polystyrene beads 4*10^7^/ml (1 μm; Polysciences, Germany), or acLDL 10 μg/ml (Invitrogen, Germany). After 72 h, the supernatants of 2-6 separate wells were pooled, spun down (750 g, 5 min) and immediately frozen until further evaluation. In two independent experiments glycosylation of APP was inhibited by adding tunicamycin or brefeldin A (both from Sigma-Aldrich, Germany) at a concentration of 10 μg/ml to unstimulated cultures 6 h, 4 h, 2 h or 0.5 h prior to cell lysis after 72 h in vitro. Cell viability was assessed morphologically and by the CytoTox 96^® ^lactate dehydrogenase assay kit (Promega, Germany). Only viable cell cultures were used for Aβ assessment.

### Aβ40-ELISA

Aβ_x-40 _in culture supernatants was quantified by ELISA according to the manufacturer's instructions (WAKO, Japan). 100 μl of undiluted supernatant were incubated over night at 4°C on the assay plate precoated with the mab BNT77 (binding AA11-28 of the Aβ peptide). Serial dilutions of synthetic Aβ_1-40 _in standard diluent buffer served as reference. Detection was performed with horseradish peroxidase-conjugated F(ab') antibody fragments (BA27) for 2 h at 4°C. The absorption at 450 nm was measured with an Infinite™M200 ELISA reader and evaluated with Magellan v6.5 software (both TECAN, Austria).

### One and two-dimensional Aβ-SDS-PAGE/immunoblot

For the analysis of Aβ peptides, the urea version of the bicine/sulfate SDS-PAGE and semi-dry western blotting (1D-Aβ-WIB) were used as previously detailed [25]. Briefly, following immunoprecipitation with the mab 1E8 (Bayer-Schering Pharma, Germany), the Aβ peptides were separated on a MiniProtean™II electrophoresis unit (BioRad, Germany). After blotting onto Immobilon™-P PVDF membranes (Millipore, Germany), immunodetection was performed with the mab 1E8. The blots were finally developed with ECL™*Advance *chemiluminescent peroxidase substrate (GE Healthcare, Germany) according to the manufacturer's protocol and analysed with the Fluor-S Max Multi-Imager and the Quantity One™v4.1 software (BioRad, Munich, Germany). All samples were run in duplicates and each gel carried a dilution series of synthetic Aβ peptides. Isoelectric focusing and second dimension Aβ-SDS-PAGE/immunoblot (2D-Aβ-WIB) was performed as described before [26]. Briefly, after immunoprecipitation with mab 1E8-activated magnetic beads (Dynal, Hamburg, Germany) the Aβ peptides were eluted and loaded onto 7 cm IPG strips (linear pH gradient, pH 4-7). Isoelectric focussing was performed with the Ettan™ IPGphor™ II System (GE Healthcare, Munich, Germany). The second dimension separation of the isoelectrically focussed Aβ peptides was performed as described above using the Hoefer electrophoresis system. Immunodetection was performed with the mab 1E8 which is specific to human Aβ peptides starting at either aspartate-1 or alanine-2 under the conditions of Aβ-WIB (Bayer Schering Pharma AG, Berlin, Germany) [27]. Synthetic peptides Aβ_1-38/40/42 _were purchased from Bachem (Weil a. Rhein, Germany). Aβ_1-37/39 _and Aβ_2-40/42 _were obtained from Biosyntan (Berlin, Germany).

### Western blot analysis of APP

For the analysis of intracellular APP, the cells were washed in PBS and lysed in RIPA-buffer (50 mM HEPES, 150 mM NaCl, 1%(v/v) Igepal, 0.5%(w/v) Na-DOC, 0.1% SDS and 1 tablet Complete Mini protease inhibitor cocktail (Roche, Germany)) on ice. After centrifugation the cell lysates were adjusted to equal protein concentrations and boiled for 5 min with the appropriate amount of fourfold concentrated sample buffer to yield a final concentration of 62.5 mM Tris/HCL pH 6.8, 2%(w/v) SDS, 10%(v/v) Glycerol; 100 mM DTT and 0.005%(w/v) bromophenolblue. Cell lysates were separated on 7.5%T/2.7%C Tris-Glycin SDS-polyacrylamide gels and blotted onto Immobilon™-P PVDF membranes (Millipore, Germany) by semi-dry transfer at a constant current of 1 mA/cm^2 ^for 60 min with 25 mM Tris, 192 mM glycine, 20%(v/v) methanol [28,29]. The membranes were blocked with 2% ECL™*Advance *blocking agent (GE Healthcare, Germany) in PBS/0.075% Tween-20 (45 min, RT) and incubated overnight at 4°C with mab 1E8. The next day the membranes were washed and incubated for 1 h at RT with a horseradish peroxidase-coupled secondary antibody (Calbiochem-Merck, Germany). After washing, the blots were developed with ECL™-*plus *chemiluminescent substrate according to the manufacturer's instructions and visualized with an Intas Imager (Intas, Göttingen, Germany). For β-actin staining the membranes were cut at approximately 60 kDa. The upper piece was stained for APP as indicated above. The lower piece was stained with an anti β-actin polyclonal antibody (Abcam, Cambridge, UK). Semiquantitative analysis of APP was performed with Quantity One™v4.1 software (BioRad, Germany).

### Statistical analysis

Data are expressed as means ± standard deviation (SD) of at least 4 separate monocyte cultures from different donors. Statistical evaluation was performed with Prism 5.0 (GraphPad Software Inc., San Diego, USA) using Kruskal Wallis test followed by Dunn's test for multiple comparison or one-way ANOVA and Dunnett's multiple comparison test. For correlation analysis Pearson's correlation coefficient was calculated. Differences were considered significant for *p *< 0.05.

## Results

### Inflammatory conditions and phagocytosis increase the release of Aβ peptides by mononuclear phagocytes

**Figure 1:**
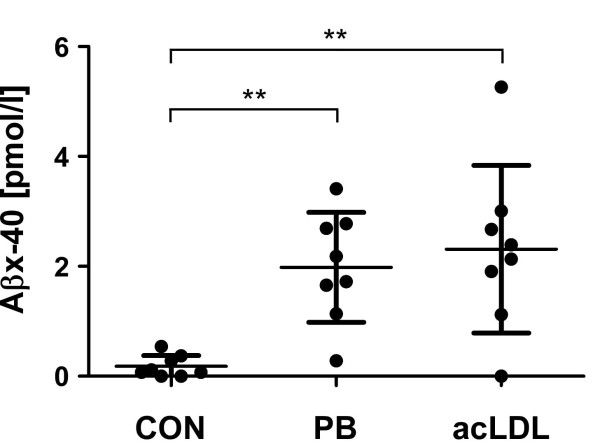
**Release of Aβ_x-40 _by mononuclear phagocytes is induced by phagocytosis of polystyrene beads or acetylated LDL**. Human mononuclear phagocytes were freshly isolated by density gradient centrifugation followed by depletion of all non phagocytes with magnetic activated cell sorting. Cells were resuspended in AIM-V medium and seeded on ultra-low attachment plates. They were left either untreated (CON) or stimulated with 4*10^7^/ml polystyrene beads (PB) or 10 μg/ml acLDL (acLDL). A β_x-40 _was assessed in supernatants after 72 h in vitro with a sandwich ELISA using the mab BNT77 (binding AA11-28 of Aβ) as the capturing antibody and horseradish peroxidase conjugated F(ab') fragments of the BA27 (specific for the C-terminus of Aβ_40_) as the detection antibody. Note the 8-11 fold increased release of Aβ_x-40 _after treatment of mononuclear phagocytes with polystyrene beads or acLDL. (p < 0,01, one-way ANOVA followed by Dunnett's multiple comparison test)

Supernatants from mononuclear phagocytes stimulated with polystyrene beads, acLDL and LPS were subjected to immunoprecipitation and 1D-Aβ-WIB. The release of total Aβ increased approximately 3-4 fold after stimulation with polystyrene beads or acLDL and reached levels of 18 and 28 pg/ml, respectively (Table 1). The concentrations of released Aβ_1-40 _were elevated to a similar extent. The latter finding was additionally confirmed by Aβ40-ELISA revealing similar elevations of Aβ_x-40 _(Fig. 1). Significant increases of Aβ secretion were also induced by LPS (Table 1). The combined stimulation of mononuclear phagocytes with polystyrene beads and LPS had an additive effect on the Aβ release (Table 1).

**Table 1:** Semiquantitative assessment of Aβ_1-4__0 _and Aβ_total _by 1D-Aβ-WIB

	CON (n = 21)	Polystyrene beads (n = 15)	acLDL (n = 10)	LPS (n = 11)	Polystyrene beads + LPS (n = 8)
**Aβ_1-40 _(pg/ml)**	1.8 ± 1.3	4.7 ± 2.1**	10.7 ± 6.5***	5.9 ± 5.1**	17.2 ± 14.9***
**Aβ_total _(pg/ml)^1^**	8.5 ± 5.9	17.9 ± 6.6**	28.4 ± 13.8***	18.1 ± 8.5*	43.8 ± 26.9***

Values are expressed as mean ± SD; *P < .05, **P < .01, ***P < .001 compared with CON (Kruskal-Wallis test followed by Dunn's multiple comparison test)

^1 ^Aβ_total _refers to Aβ_1-38/39/40/42, _Aβ_2-40 _and Aβ_2-40_^a^

### Phagocytosis but not LPS increase the proportion of N-terminally truncated Aβ peptides released by mononuclear phagocytes

**Figure 2:**
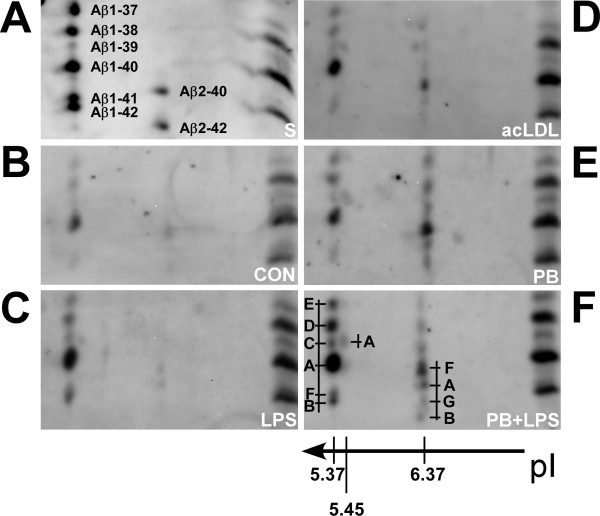
**Release of N-terminally truncated Aβ-peptides is induced by polystyrene beads and acetylated LDL but not by LPS**. CD14^+ ^monocytes were negatively isolated by MACS and kept in serum-free AIM-V medium in ultra low binding culture vessels. Following stimulation for 72 h cell culture supernatants were collected and subjected to immunoprecipitation with mab 1E8-preactivated magnetic beads. Released Aβ peptides were assessed by isoelectric focusing on linear IPG strips (pH4-7) and subsequent Aβ-SDS-PAGE (2D-Aβ-WIB). (**A**) shows the separation of the following synthetic standard Aβ peptides: Aβ_1-37_, Aβ_1-38_, Aβ_1-39_, Aβ_1-40_, Aβ_1-42 _as well as Aβ_2-40 _and Aβ_2-42_. For a better orientation, synthetic Aβ_1-37_, Aβ_1-38_, Aβ_1-39_, Aβ_1-40_, Aβ_1-42 _were additionally separated one-dimensionally on the right side of each gel. The following figures show 2D-Aβ-WIBs from supernatants of untreated cultures (**B**) or cultures stimulated with LPS 10 ng/ml (**C**), acLDL 10 μg/ml (**D**) and 1 μm carboxylated polystyrene beads 4*10^7^/ml (**E**) that were processed as described above. In (**F**), the LPS stimulus was combined with polystyrene beads at the concentrations used in (**C**) and (**E**), respectively. Distinct Aβ reactive spots are indicated by characters and by the respective pI values. Note the proportional increase of released N-truncated Aβ peptides migrating at pI 6.37 upon phagocytosis (**D**, **E**, **F**).

To resolve the highly complex pattern of Aβ peptides secreted by mononuclear phago-cytes and to study in detail the specific effects of each stimulus on different Aβ variants, two-dimensional 2D-Aβ-WIB was performed (Fig. 2). It appeared that the increases in total Aβ release after different stimuli were accompanied by substantial alterations in the relative abundance of the different Aβ variants. In untreated control cells, Aβ_1-40 _accounted for ~64% of total Aβ while this relative proportion was significantly lower in cells treated with polystyrene beads, acLDL or LPS. This was accompanied by increased proportions of several Aβ peptides with more alkaline pI values representing N-terminally truncated Aβ_2-x _variants (Table 2). In contrast, the relative amount of secreted Aβ_1-42 _remained constant under all tested stimulation paradigms.

**Table 2:** Proportions of single Aβ peptide species secreted by non-adherent mononuclear phagocytes stimulated with polystyrene beads, acLDL or LPS (2D-Aβ-WIB, mab 1E8)

			CON (n = 5)	Polystyrene beads (n = 5)	acLDL (n = 4)	LPS (n = 4)	Polystyrene beads + LPS (n = 4)
**Spot**	**pI**	**Aβ identity proposed**	**% of total Aβ**				

**5.37-A**	5.37	Aβ_1-40_	63.9 ± 3.8	30.0 ± 7.0***	37.5 ± 6.5***	49.2 ± 9.5*	42.0 ± 12.0**
**5.37-B**	5.37	Aβ_1-42_	3.7 ± 1.9	2.8 ± 0.8	3.2 ± 0.2	4.0 ± 0.1	3.3 ± 0.2
**5.37-C**	5.37	Aβ_1-39_	1.8 ± 0.1	4.0 ± 0.8***	4.9 ± 0.4***	4.8 ± 0.6***	4.2 ± 0.9***
**5.37-D**	5.37	Aβ_1-38_	9.6 ± 1.9	12.4 ± 1.1	13.1 ± 1.3*	13.4 ± 1.5*	11.9 ± 2.5
**5.37-E**	5.37	Aβ_1-37_	5.7 ± 1.2	7.2 ± 1.2	8.5 ± 1.6	7.3 ± 1.2	6.8 ± 2.3
**5.37-F**	5.37	Aβ_1-41_	2.9 ± 1.6	2.7 ± 0.6	3.0 ± 0.3	3.7 ± 0.2	2.5 ± 0.7

**6.37-A**	6.37	Aβ_2-40_	1.7 ± 0,7	6.0 ± 1.1**	4.2 ± 0.6	2.8 ± 1.1	6.4 ± 3.1***
**6.37-B**	6.37	Aβ_2-42_	0.6 ± 0.4	4.3 ± 1.2***	3.7 ± 0.8**	2.5 ± 1.2*	3.5 ± 1.5**
**6.37-F**	6.37	n. i., Aβ_2-40_^a^	8.4 ± 5.3	20.1 ± 5.9**	11.4 ± 1.7	4.4 ± 1.5	10.0 ± 2.3
**6.37-G**	6.37	n. i.	0.5 ± 0.4	4.7 ± 1.2***	3.8 ± 0.9***	2.6 ± 1.2*	3.4 ± 1.6**

**5.45-A**	5.45	n.i., Aβ_2-40_^b^	1.3 ± 0.9	5.9 ± 1.3**	6.7 ± 0.7***	5.3 ± 2.0**	6.0 ± 2.9**

Values are expressed as mean ± SD; *P < .05, **P < .01, ***P < .001 compared with CON (one-way ANOVA followed by Dunnett's multiple comparison test)

n. i. - not identified

Most interestingly, the proportion of N-terminally truncated Aβ_2-x _peptides increased several-fold upon phagocytic stimuli. After stimulation with acLDL, Aβ_2-x _species together represented almost 30% of Aβ_total _(Fig. 2D, Table 2). Even higher proportions of more than 40% of Aβ_total _were observed after stimulation with polystyrene beads (Fig. 2E, Table 2). In comparison, Aβ_2-x _species accounted for only ~12% of Aβ_total _in control cultures (Fig. 2B, Table 2). The proportion of N-truncated Aβ species in LPS-stimulated mononuclear phagocyte cultures was only marginally increased and the overall Aβ pattern resembled that found in unstimulated controls with Aβ_1-x _species accounting for more than 80% of Aβ_total _(Fig. 2C, Table 2). The combination of an inflammatory (LPS) with a phagocytic stimulus (polystyrene beads) integrates the effect of the two individual treatments with a strong release of both, Aβ_1-x _and Aβ_2-x _species (Fig. 2F).

**Figure 3:**
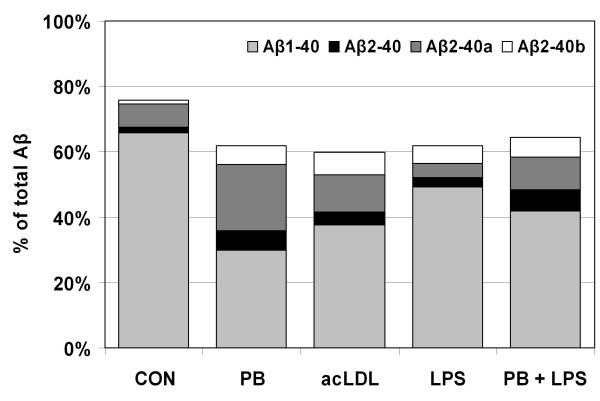
**Stimulation with acLDL, polystyrene beads, and LPS induces shifts in proportions of Aβ40 variants**. The graph depicts the sum of proportions of the 4 different Aβ40 variants after stimulation with LPS, acLDL and polystyrene beads. Note that this sum decreases upon stimulation but remains almost unaffected by the different stimuli accounting for about 60% of Aβ_total_. Nonetheless, strong shifts are visible within single Aβ_x-40 _variants resulting in a stimulus-specific signature. Especially the proportion of the Aβ peptide migrating at spot 6.37-F - presumably a variant of Aβ_2-40 _(Aβ_2-40_^a^) - shows strong stimulus-dependent shifts.

A stimulus-specific signature of secreted Aβ peptides can be deduced from the distribu-tion of the four Aβ40 peptides previously designated as Aβ_1-40_, Aβ_2-40_, Aβ_2-40_^a^, Aβ_2-40_^b ^[24]. The identity of the peptides was concluded on the basis of co-migration with synthetic Aβ peptides and their detection with N- and C- terminally specific antibodies [24]. The peptides display isoelectric points of approximately 5.37, 6.37, and 5.45 and their positions after 2D-Aβ-WIB separation as well as a scheme to indicate the terminology used in Table 2 are indicated in Fig. 2F. The sum of proportions of the four Aβ40 peptides remained stable at about 60% of Aβ_total _independently of the stimulus (Table 2, Fig. 3). Nonetheless, characteristic shifts in the relative proportion of individual Aβ40 variants were observed. Most obviously a prominent increase of Aβ_2-40_^a ^at the expense of Aβ_1-40 _was visible in cultures stimulated with polystyrene beads.

### Inflammatory conditions and phagocytosis increase protein levels of APP

**Figure 4:**
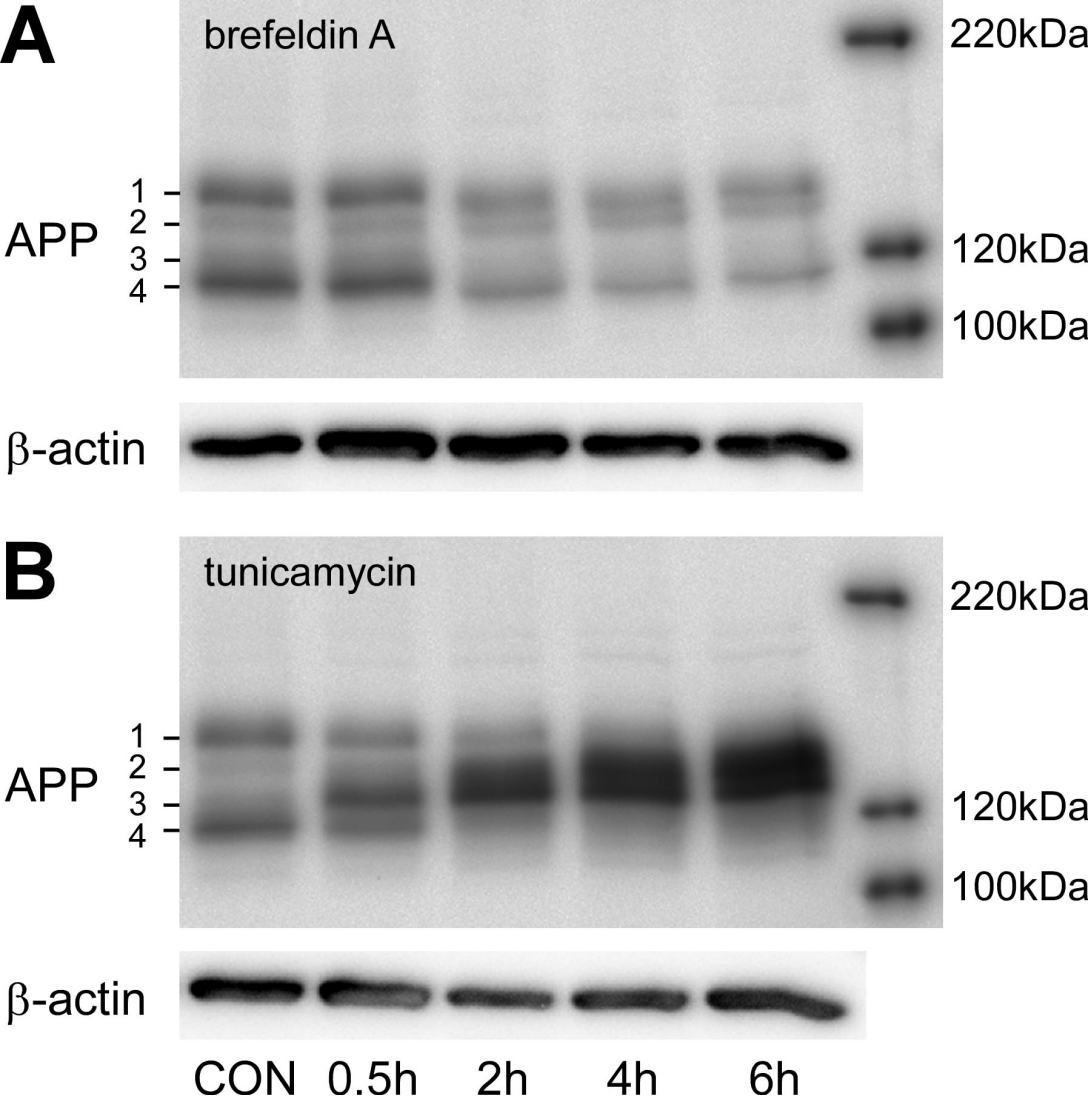
**Shifts in full length APP patterns induced by the glycosylation inhibitors tunicamycin and brefeldin A in mononuclear phagocyte cultures**. Human mononuclear phagocytes were isolated as indicated and left unstimulated on ultra-low attachment plates for 3 days. 6 h, 4 h, 2 h or 0.5 h prior to the lysis of the cells, tunicamycin or brefeldin A were added in a concentration of 10 μg/ml each. Cells were lysed in RIPA buffer and APP expression was analysed by separation on 7.5% SDS-PAGE, subsequent blotting on PVDF-membranes and staining with the 1E8 monoclonal antibody. Staining of β-actin served as a loading control. The right hand side shows a molecular weight standard. Note the slight shift in molecular weight after brefeldin A treatment and the decrease of band APP1 corresponding to mature APP and the increased amounts of the bands APP2 and APP3 after treatment with Tunicamycin.

To examine the effects of LPS and phagocytic stimuli on the APP metabolism we assessed the protein levels of APP in mononuclear phagocytes. Electrophoretic separation of APP holoprotein from unstimulated cells revealed a pattern of 4 prominent APP-reactive bands with molecular weights of approximately 120-140 kDa presumably corresponding to differently maturated forms of APP_751 _and APP_770 _(Fig. 4). Inhibition of O-glycosylation with brefeldin A resulted in the time-dependent reduction of the protein level of total APP and a slight decrease in the apparent molecular weights of APP bands 1 and 4 (Fig. 4A). In contrast, inhibition of N-glycosylation with tunicamycin induced a strong increase in total APP and a strong increase of the lighter bands APP2/APP3 at the expense of the heaviest band APP1 (Fig. 4B). Taken together the results indicate that only APP1 accounts for fully glycosylated, mature APP.

**Figure 5:**
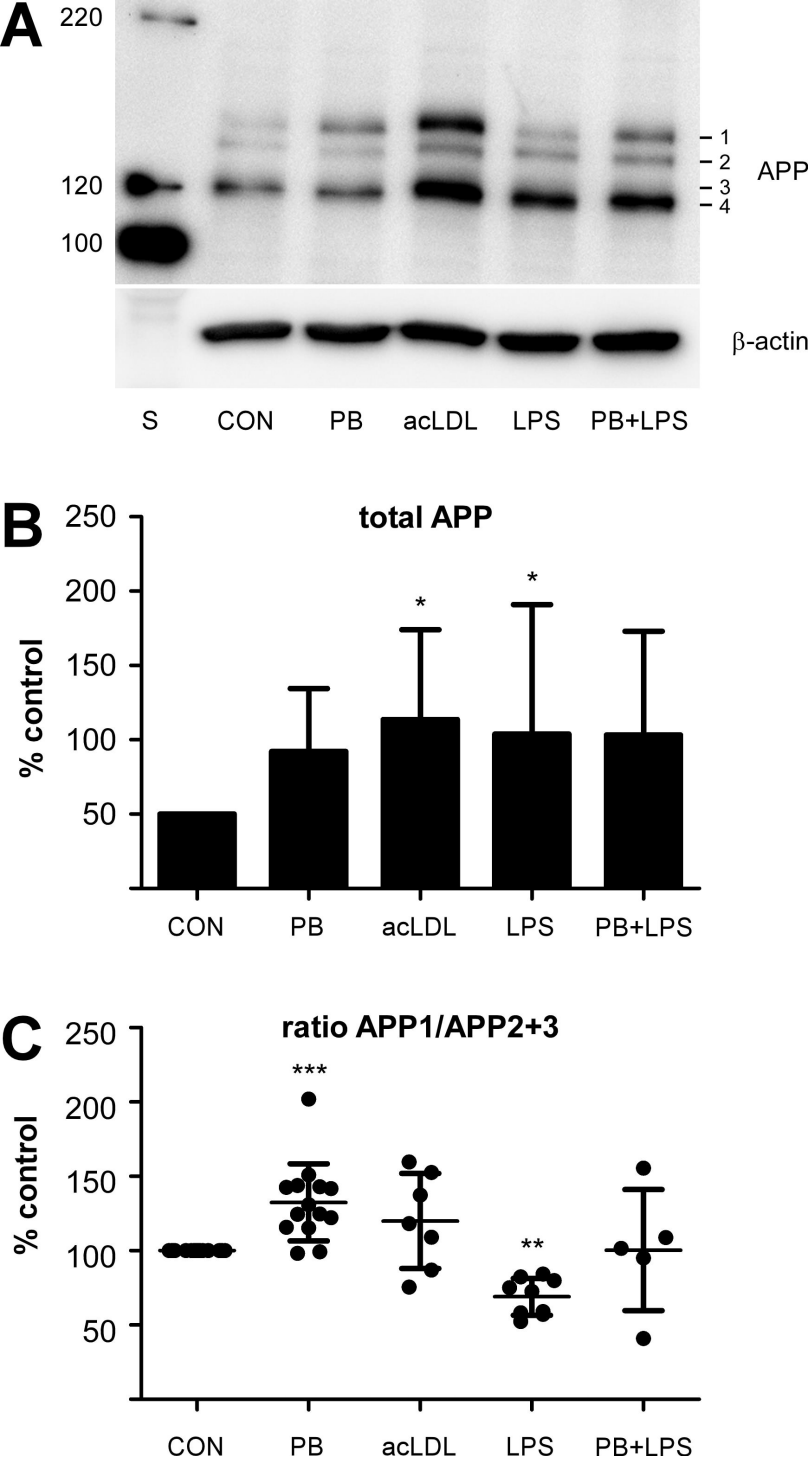
**Shifts in full length APP patterns induced by acLDL, polystyrene beads, and LPS**. Human mononuclear phagocytes were isolated and cultivated as indicated and left unstimulated (CON) or stimulated with 4*10^7^/ml polystyrene beads (PB), 10 μg/ml acLDL (acLDL), 10 ng/ml LPS (LPS) or a combination of polystyrene beads and LPS (PB+LPS). (**A**) shows a representative SDS-PAGE/immunoblot of whole cell lysates from human mononuclear phagocytes after 72 h in vitro immunoreacted with the 1E8 antibody. Staining of the same western blots with a polyclonal antibody specific for β-actin served as a loading control. (**B**) Semiquantitative assessment of the changes in expression of total APP (APP1+ APP2+APP3+APP4) compared to control. (**C**) Shifts in the ratio APP1:APP2+3 after application of the different stimulation paradigms. Values are expressed as mean ± SD. *P < .05, **P < .01, ***P < .001 compared with CON (one-way ANOVA followed by Dunnett's multiple comparison test). Note the increase in total APP expression under all conditions as well as the increased proportion of APP1 (mature APP) after stimulation with polysterene beads or acLDL.

In a further step, we analysed the expression of APP after stimulation with either acLDL, poly-styrene beads, LPS or LPS together with polystyrene beads (Fig. 5). Although statistically significant only for the stimulation with acLDL and LPS, the expression of total APP increased approximately twofold in all stimulation paradigms compared to control (Fig. 5A, B).

### Phagocytosis but not LPS increases the levels of mature/glycosylated APP

On the basis of the experiments with brefeldin A and tunicamycin, the ratio APP1:APP2+3 was calculated to monitor the effects of stimulation on the maturation/glycosylation state of APP (Fig. 5C). The ratio APP1:APP2+3 increased significantly upon phagocytosis of polystyrene beads as compared to control (p < 0.001) and a similar trend was observed after stimulation with acLDL. These data indicate an increased protein level of mature APP. In contrast, the ratio APP1:APP2+3 decreased significantly upon stimulation with LPS (p < 0.01). In cultures stimulated with LPS together with polystyrene beads the ratio APP1:APP2+3 remained at the control level.

**Figure 6:**
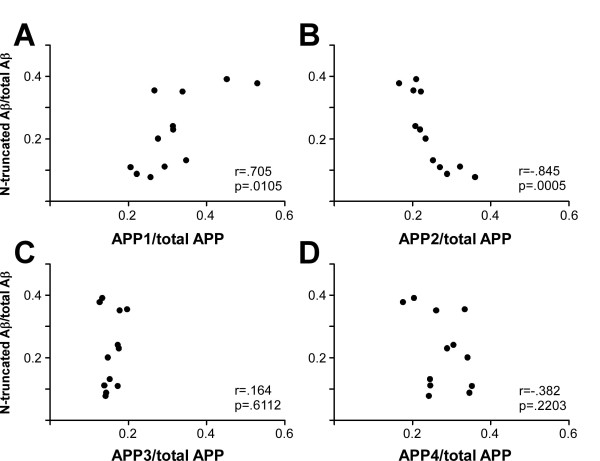
**Expression of mature APP correlates with the release of N-terminally truncated Aβ peptides**. For the correlation analysis 12 cultures (1× CON, 4× PB, 2× acLDL, 3× LPS, 2× PB+LPS) were analysed simultaneously for intracellular APP and released Aβ-peptides by APP-SDS-PAGE and 2D-Aβ-WIB, respectively. Pearson's correlation coefficient (r) and the respective p-value were calculated for the association of the relative amounts of APP band 1 (APP1), band 2 (APP2), band 3 (APP3), and band 4 (APP4) with the relative amounts of N-terminally truncated Aβ peptides migrating at the isoelectric point pI 6.37 (N-truncated Aβ). The relative amount of single APP-bands was determined by dividing the signal intensity of the respective band by the sum of the signal intensities of all four visible APP bands. For the relative amount of N-truncated Aβ the sum of all visible Aβ isoforms migrating at pI 6.37 (Aβ_2-40_, Aβ_2-42_, Aβ_2-40_^a ^and the Aβ peptide at spot 6.37-G) were divided by the sum of all quantified Aβ-peptide spots. The proportion of N-terminally truncated Aβ peptides correlates positively with mature APP (APP1) and negatively with APP2.

The proportion of released N-truncated Aβ peptides (sum of Aβ variants migrating at pI 6.37) correlated positively with the proportion of mature APP, i.e. APP band 1 (Pearson's r = .705, p = .0105), while the correlation with the immature APP band 2 was negative and even stronger (Pearson's r = - .845, p = .0005) (Fig. 6). These data suggest a relationship between APP glycosylation/maturation and the generation of N-truncated Aβ peptide variants.

## Discussion

A direct role of the LPS receptor (CD14/TLR4) in the phagocytosis of fibrillar Aβ was suggested because of its upregulation in β-amyloid plaque-associated microglia and its interaction with fibrillar Aβ [23,30]. In the current study, we observed that both, phagocytosis and activation of the LPS receptor induced a twofold increase in APP expression and a several-fold increase of Aβ_total _and Aβ_1-40 _secretion by human mononuclear phagocytes. In agreement with our present findings in primary human mononuclear phagocytes, increased levels of APP upon activation or differentiation were also observed in earlier studies in microglia and monocytes, respectively [13,31]. Similarly, an increase in the secretion of monomeric Aβ peptides has been observed in immortalized BV-2 mouse microglial cells upon stimulation with LPS [32]. In contrast to mononuclear phagocytes stimulated by phagocytosis, the Aβ release signature induced by activation of the LPS receptor is characterized by only minute proportions of N-truncated Aβ species and a high abundance of Aβ_1-x_, i.e. Aβ species starting with aspartate-1. The signalling induced by LPS/CD14/TLR4 occurs mainly via the NF-κB pathway. It has been shown that members of the NF-κB family of transcriptional control proteins are critical regulators of APP gene expression and that NF-κB inhibitors decrease both Aβ_1-40 _and Aβ_1-42 _production [33,34].

Unexpectedly, the stimulation of mononuclear phagocytes with both polystyrene beads and ac-LDL increased the protein levels of APP and the release of Aβ peptides as well but elicited a different Aβ pattern with increased proportions of N-truncated Aβ species (Aβ_2-x_). The expression of APP is associated with high membrane fusion activity [35]. Membrane rearrangement following phagocytosis includes delivery of new membrane to the sites of particle ingestion by the unwrinkling of surface folds and the delivery of endomembranes by focal exocytosis [36-38]. Membrane sources in mammalian phagocytes are recycling endosomes and late endosomes but also the release of lysosomes has been reported [38,39]. Furthermore, APP is involved in monocyte adhesion to the extracellular matrix or to other cells [40]. Increased APP expression during phagocytosis might therefore be important for the function of innate immunity.

APP deficient in core N-glycosylation is processed intracellularly, whereas only the mature form is inserted into the cellular membrane resulting in the secretion of soluble APP fragments [15-17]. After phagocytic stimuli elevated proportions of released N-truncated Aβ peptides are accompanied by increased levels of mature APP. This might indicate a site of Aβ generation that differs from that affected by stimulation with LPS. A possible explanation for this observation is the finding that processing of APP at the plasma membrane seems to promote the generation of N-terminally truncated Aβ peptides [41].

High percentages of N-truncated or posttranslationally modified Aβ peptide species such as 3-pyroglutamate Aβ_N3-pE _and Aβ_2-x _variants have been found in neuritic and vascular β-amyloid plaques [8,9]. Compared to mouse models of AD a much higher degree of N-terminal degradation of Aβ peptides was found in human samples [42]. Their (sub)cellular origin and their role in amyloidogenesis *in vivo *is still only partly understood but it was shown that aggregation of Aβ peptides *in vitro *is enhanced by N-terminal deletions [43]. It was further reported that Aβ40 oligomerization, in contrast to Aβ42 oligomerization, was particularly sensitive to truncations of the N terminus [44].

Upon stimulation the relative proportion of the most abundant form of Aβ, Aβ_1-40_, decreased relative to total Aβ by up to 50%. These shifts within the Aβ release signature were particularly visible when only 4 Aβ variants ending at valine-40 (Aβ_x-40_) were taken into consideration [24]. 2D-Aβ-WIB and semiquantitative assessment of the proportions of specific Aβ variants revealed pronounced and stimulus-specific shifts towards N-truncated or otherwise modified variants of Aβ_2-40 _at the expense of Aβ_1-40_. The sum of all these Aβ40 variants remained surprisingly stable. In contrast to Aβ_x-40_, the proportions of Aβ_x-42 _remained almost unaffected by the different stimuli. In many cell types, the production of Aβ peptides occurs predominantly in the endosomal/lysosomal compartment and different pools of Aβ peptides with different distributions of single Aβ species, i.e. Aβ_1-40 _and Aβ_1-42_, have been reported [11,45]. Therefore, the shifts in Aβ40 variants might reflect a release from different intracellular pools of Aβ peptides.

## Conclusions

The present data show that activation of the LPS receptor and of scavenger receptor-associated signalling pathways by acLDL and polystyrene beads increases the cellular protein levels of APP and the secretion of Aβ peptides by human mononuclear phagocytes. The expression of scavenger receptors and CD14 - which both bind fibrillar Aβ- is increased in AD. Therefore, it might be speculated that the presence of fibrillar Aβ itself increases the release of monomeric Aβ variants by human phagocytes and thereby initiates a vicious cycle. Further work is necessary to assess how stimulus-specific shifts in the Aβ release signature contribute to amyloidogenesis *in vivo*.

## Abbreviations

AD: Alzheimer's disease; APP: β-amyloid precursor protein; SR: scavenger receptors; CON: control; PB: polystyrene beads; ACLDL: acetylated LDL; OXLDL: oxidized LDL; AGE: advanced glycation endproducts; LPS: lipopolysaccharide; PBMC: peripheral blood mononuclear cells; MAB: monoclonal antibody; MACS: magnetic activated cell sorting; Aβ-WIB: Aβ-SDS-PAGE western immunoblot; TLR4: Toll-like receptor 4.

## Competing interests

The authors declare that they have no competing interests.

## Authors' contributions

PS and JMM designed and conducted the study, analyzed data, prepared the figures and wrote the manuscript. MH participated in study design and carried out the flow cytometry assays. HWK and JW provided expertise in interpretation and analysis of data obtained from Aβ-SDS-PAGE western immunoblot. Western blots for APP assessment were performed by AS. HWK, PL, JK and JW reviewed the manuscript extensively and provided constructive comments to improve the quality of the manuscript. All authors read and approved the final manuscript.
